# Epidemics of Influenza and Pediatric Diseases Observed in Infectious Disease Surveillance in Japan, 1999-2005

**DOI:** 10.2188/jea.17.S14

**Published:** 2008-01-30

**Authors:** Akiko Ohta, Yoshitaka Murakami, Shuji Hashimoto, Masaki Nagai, Miyuki Kawado, Michiko Izumida, Yuki Tada, Mika Shigematsu, Yoshinori Yasui, Kiyosu Taniguchi

**Affiliations:** 1 Department of Public Health, Saitama Medical University Faculty of Medicine.; 2 Department of Health Science, Shiga University of Medical Science.; 3 Department of Hygiene, Fujita Health University School of Medicine.; 4 Infectious Disease Surveillance Center, National Institute of Infectious Diseases.

**Keywords:** Influenza, Human, Communicable Diseases, Disease Outbreaks, Sentinel Surveillance

## Abstract

**BACKGROUND:**

A method for determining epidemics in small areas from the sentinel surveillance data has been proposed and applied in the National Epidemiological Surveillance of Infectious Diseases (NESID) in Japan. We observed epidemics of influenza and 11 pediatric diseases by the method in the NESID in Japan during 1999-2005.

**METHODS:**

We assumed that an epidemic in a public health center area began in a week when the number of cases reported to the NESID per sentinel clinic and hospital in the area in the week exceeded a given value, and that the epidemic ended when the number was lower than another given value. The proportion of public health center areas with epidemics (epidemic area proportion) by week in fiscal 1999-2005 was calculated. Total public health center area-weeks observed were about 30,000 each year.

**RESULTS:**

The mean epidemic area proportion in the 7 years was 6.0% for influenza and 0.2-7.4% for pediatric diseases. The proportion increased in pharyngoconjunctival fever and group A streptococcal pharyngitis, decreased in measles and was less than 1.0% in pertussis and rubella. In influenza, the height of the peak in the weekly epidemic area proportion varied between 6 and 90% in the 7 years and the week of the peak varied widely. In some pediatric diseases, the height of the peak varied, while the week of the peak was relatively constant.

**CONCLUSION:**

The frequency and temporal change were described in the epidemics of influenza and pediatric diseases in public health center areas from the NESID data in Japan, 1999-2005.

Epidemics of infectious diseases generally begin in small areas and subsequently spread widely. For the control and prevention of epidemics in a large area (such as an entire country), observing epidemics in small areas (such as municipalities) is essential, and understanding their characteristics, such as frequency and temporal change, is important.

In many countries, sentinel surveillance of infectious diseases is conducted^1^^-^^10^ for detecting the epidemic in its early stage. A method has been proposed for determining the epidemics in small areas from the sentinel surveillance data.^11^ The method is based on the large number of cases reported in a given week per sentinel clinics and hospitals as mentioned below. In Japan, this method has been applied^11^^-^^15^ as an alert system for epidemics of influenza and pediatric diseases such as measles in public health center areas since 1999 in the National Epidemiological Surveillance of Infectious Diseases (NESID). Although the characteristics of those epidemics of influenza and pediatric diseases in public health center areas in 1993-1997^11^ and those of influenza in 1999^13^ were reported, the epidemics of recent years have not been described in detail.

In the present study, we described the frequency and temporal change of the epidemics of influenza and pediatric diseases in public health center areas observed by the proposed method for determining epidemics in the NESID in Japan, 1999-2005.

## METHODS

### Surveillance of Infectious Diseases in Japan

The NESID in Japan was enacted by the Law Concerning the Prevention of Infectious Diseases and Medical Care for Patients of Infections in 1999. It has been described in detail elsewhere.^10^ The influenza and pediatric disease surveillance system is comprised of sentinel clinics and hospitals for pediatric diseases (about 3,000 pediatric facilities throughout Japan) and sentinels for influenza (3,000 pediatric facilities as mentioned above plus about 2,000 internal medicine facilities). The numbers of sentinels in public health center areas are approximately proportional to their population sizes. The populations covered by public health centers vary widely in size from less than 30,000 to more than 250,000. The sentinels were recruited on a voluntary basis to report the number of cases of influenza and pediatric diseases weekly to public health centers. Reports from public health centers to the local government (prefecture) and the Ministry of Health, Labour and Welfare of Japan are made through an on-line computer network.

### Surveillance Data and Method for Determining Epidemics

We used the data from the NESID in Japan for the fiscal years 1999-2005. Fiscal 1999, for example, means the period from April 1999 through March 2000. The numbers of cases of influenza and pediatric diseases per sentinel clinics and hospitals weekly reported in the public health center area were used as indices for the analysis. This analysis targeted influenza and other 11 diseases as shown in Table 1. The public health center areas greatly changed in 1999-2003. When public health center areas were combined or divided during this period, we combined them into one area for the sake of easier analysis. The number of public health center areas for the analysis was 568 in 1999-2003, 547 in 2004, and 545 in 2005.

**Table 1.** Critical values for determining onset and end of epidemic.

Disease	Critical value for epidemic	

	onset	end
Influenza	30.0	10.0
Pharyngoconjunctival fever	2.0	0.1
Group A streptococcal pharyngitis	4.0	2.0
Infectious gastroenteritis	20.0	12.0
Chickenpox	7.0	4.0
Hand-foot-mouth disease	5.0	2.0
Erythema infectiosum	2.0	1.0
Pertussis	1.0	0.1
Rubella	1.0	0.1
Herpangina	6.0	2.0
Measles	1.5	0.5
Mumps	6.0	2.0

Numbers in table indicate no. of cases per week per sentinel clinic and hospital.

In order to determine the occurrence of epidemics in a public health center area, the proposed method was applied as follows. We assumed that an epidemic in a public health center area began in a week when the number of cases per sentinel clinics and hospitals in the area in the week exceeded a certain value for the onset of an epidemic, and that the epidemic ended when the number was lower than another critical value for the end of the epidemic. Table 1 shows critical values for the onset and the end of the epidemic. The critical value was determined according to distribution of the number of cases per week per sentinel clinics and hospitals using the surveillance data.^11^^,^^12^

### Method of Analysis

We observed the occurrences of epidemics of influenza and 11 pediatric diseases in public health center area weekly during fiscal year of 1999-2005, based on the method for determining epidemics mentioned above. Total public health center area-weeks observed, which were the number of public health center areas times weeks in each fiscal year from 1999 through 2005, were 30,104, 29,536, 29,522, 29,468, 29,484, 28,965, and 27,795, respectively.

To evaluate the frequency of epidemics, the proportion of public health center areas with epidemics (denoted as epidemic area proportion) was calculated. To describe the temporal change of epidemics, the epidemic area proportion by week was also calculated and figured. We attempted to use some indices of temporal change of epidemics for the diseases with a clear seasonal pattern, including height and week of peak, the first week, the last week, and the duration of the elevation in the weekly epidemic area proportion. The elevation means that the epidemic area proportion is over the 5% level. We considered that the elevation continued even if the epidemic area proportion was less than 5% in an exceptional few weeks. We took the height of the peak in the epidemic area proportion as an index of the geographical spread of the epidemic nationwide, the week of its peak as an index of time or season of the epidemic, and the duration of the elevation over the 5% level of the epidemic area proportion as an index of temporal accumulation of the epidemic.

## RESULTS

Table 2 shows the number of cases per week per sentinel clinics and hospitals in fiscal years of 1999-2005. The number of influenza cases varied between 1.10 and 5.85 during the 7 years. Among 11 pediatric diseases, the number of cases contracting pertussis, rubella and measles was low, but high in infectious gastroenteritis.

**Table 2.** Number of cases per week per sentinel clinic and hospital in fiscal 1999-2005.

Disease	Fiscal year						

	1999	2000	2001	2002	2003	2004	2005
Influenza	3.64	1.10	2.78	5.09	3.19	5.85	4.11
Pharyngoconjunctival fever	0.08	0.15	0.15	0.10	0.29	0.37	0.35
Group A steptococcal pharyngitis	0.83	1.11	0.98	0.95	1.19	1.21	1.33
Infectious gastroenteritis	5.56	5.76	5.54	5.56	5.93	5.75	5.95
Chickenpox	1.59	1.88	1.61	1.66	1.67	1.49	1.55
Hand-foot-mouth disease	0.35	1.35	0.79	0.58	1.08	0.59	0.54
Erythema infectiosum	0.16	0.28	0.44	0.31	0.25	0.28	0.26
Pertussis	0.02	0.02	0.01	0.01	0.01	0.01	0.01
Rubella	0.02	0.02	0.02	0.02	0.02	0.02	0.01
Herpangina	1.02	0.94	0.91	0.71	0.94	0.66	0.93
Measles	0.06	0.18	0.18	0.07	0.04	0.01	0.00
Mumps	0.58	1.12	1.61	0.92	0.52	0.93	1.27

Table 3 shows the epidemic area proportion in total public health center area-weeks in each fiscal year of 1999-2005. The epidemic area proportion of influenza varied widely between 0.4 and 10.8% in the 7 years, with a mean of 6.0%. In 11 pediatric diseases, the mean of proportions in the 7 years was between 0.2 and 7.4%. The proportion of pharyngoconjunctival fever and group A streptococcal pharyngitis increased. The proportion of measles decreased and was less than 0.1% since 2004. The proportion of pertussis and rubella was less than 1.0%. The proportion of chickenpox, hand-foot-mouth disease, erythema infectiosum and mumps varied widely in the 7 years, while that of infectious gastroenteritis and herpangina was relatively constant.

**Table 3.** Proportion of public health center areas with epidemic in total public health center area-weeks in fiscal 1999-2005.

Disease	Fiscal year						

	1999	2000	2001	2002	2003	2004	2005
Influenza	5.5	0.4	3.3	9.8	5.6	10.8	6.3
Pharyngoconjunctival fever	0.9	3.0	3.5	1.8	7.2	10.6	8.7
Group A steptococcal pharyngitis	4.6	7.7	6.5	5.3	8.3	9.2	10.4
Infectious gastroenteritis	6.5	7.0	6.1	5.5	6.2	5.5	6.0
Chickenpox	2.8	4.3	2.6	2.9	3.1	2.0	2.1
Hand-foot-mouth disease	1.6	10.5	5.2	2.9	8.1	3.3	2.4
Erythema infectiosum	1.7	3.1	6.5	4.3	2.1	3.3	2.6
Pertussis	0.3	0.4	0.1	0.1	0.1	0.1	0.0
Rubella	0.7	0.3	0.3	0.4	0.6	0.7	0.0
Herpangina	7.8	6.2	6.6	4.3	7.3	4.2	6.6
Measles	1.2	3.8	4.6	1.3	0.5	0.0	0.0
Mumps	1.5	4.3	8.9	4.2	1.1	2.6	3.9

Figures in table indicate proportion (%).

Figures 1 to 12 shows the epidemic area proportion of influenza and 11 pediatric diseases by week in fiscal years of 1999-2005, respectively. The seasonal pattern with one peak in a year was observed in the epidemic area proportions of influenza and many pediatric diseases. Two peaks in one year were observed in those of infectious gastroenteritis. Low or no peak was observed in those of pertussis, rubella, group A streptococcal pharyngitis and mumps.

**Figure 1.**
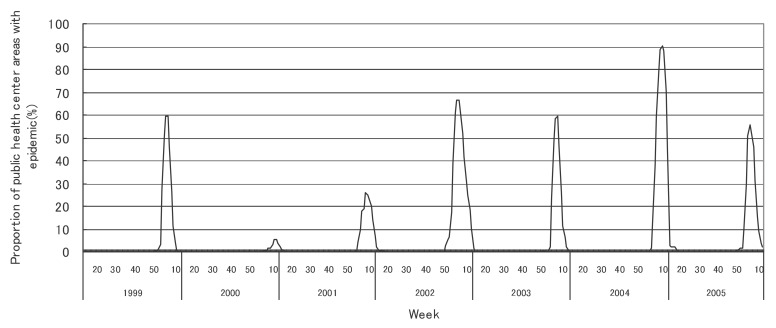
Proportion of public health center areas with epidemic of influenza by week in fiscal 1999-2005.

**Figure 2.**
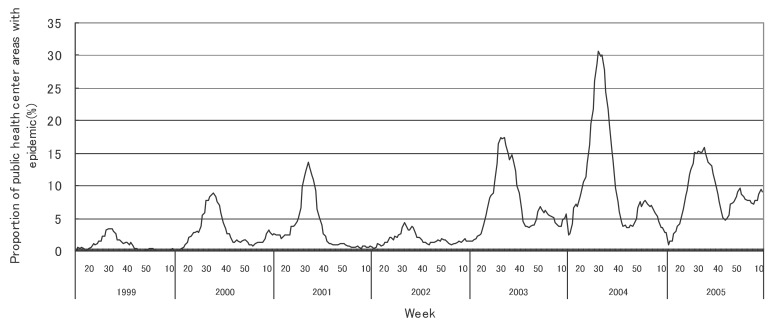
Proportion of public health center areas with epidemic of pharyngoconjunctival fever by week in fiscal 1999-2005.

**Figure 3.**
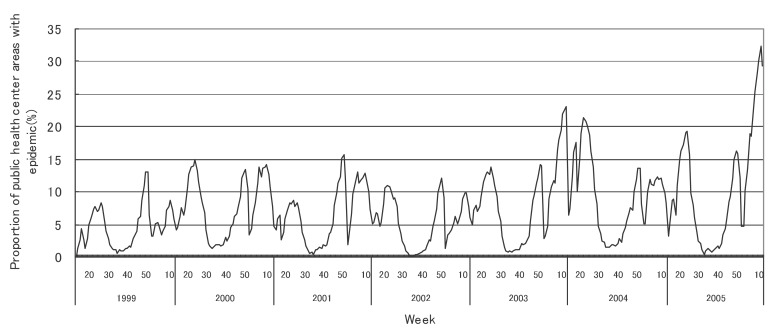
Proportion of public health center areas with epidemic of group A streptococcal pharyngitis by week in fiscal 1999-2005.

**Figure 4.**
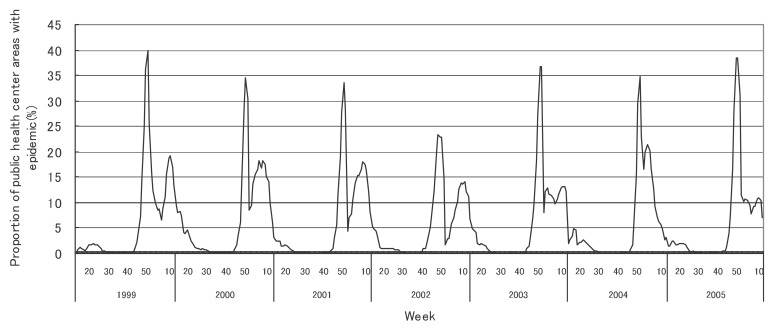
Proportion of public health center areas with epidemic of infectious gastroenteritis by week in fiscal 1999-2005.

**Figure 5.**
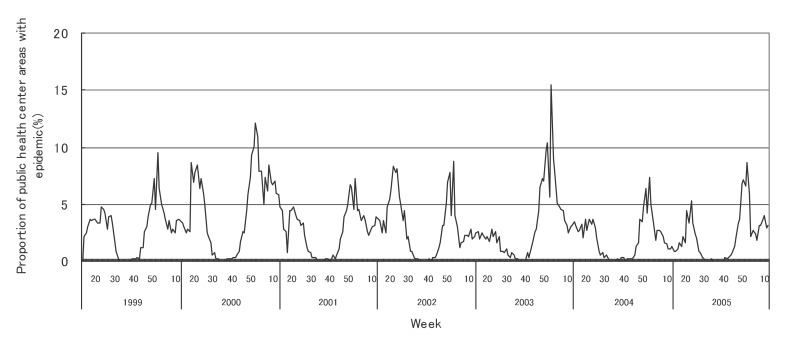
Proportion of public health center areas with epidemic of chickenpox by week in fiscal 1999-2005.

**Figure 6.**
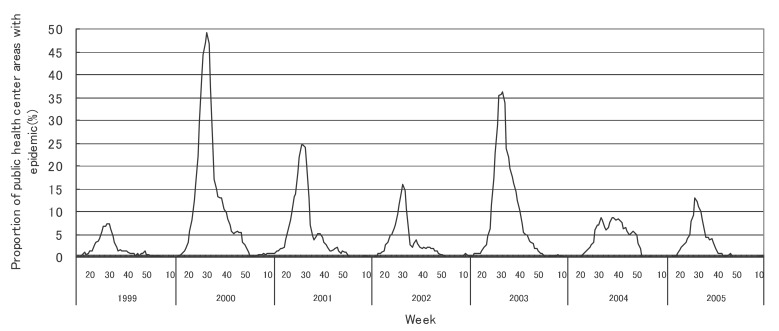
Proportion of public health center areas with epidemic of hand-foot-mouth disease by week in fiscal 1999-2005.

**Figure 7.**
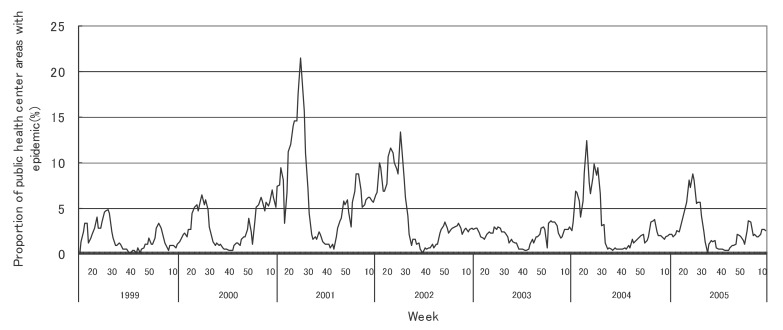
Proportion of public health center areas with epidemic of erythema infectiosum by week in fiscal 1999-2005.

**Figure 8.**
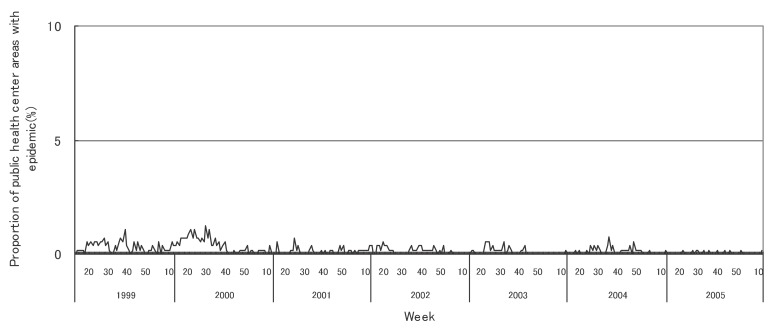
Proportion of public health center areas with epidemic of pertussis by week in fiscal 1999-2005.

**Figure 9.**
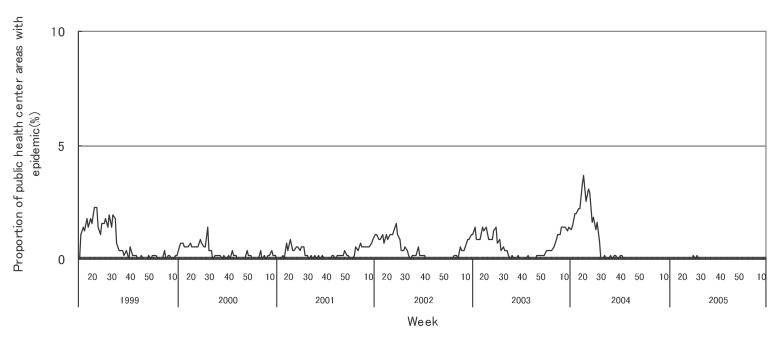
Proportion of public health center areas with epidemic of rubella by week in fiscal 1999-2005.

**Figure 10.**
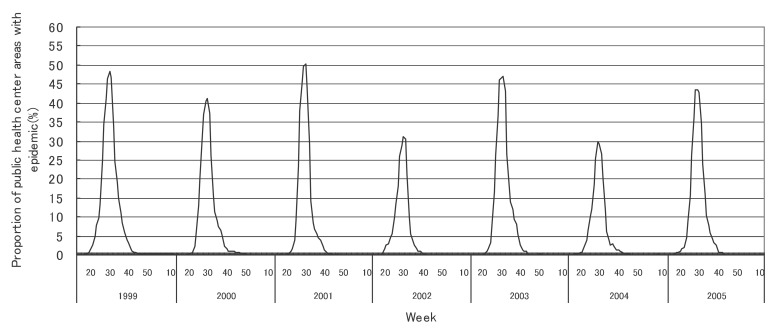
Proportion of public health center areas with epidemic of herpangina by week in fiscal 1999-2005.

**Figure 11.**
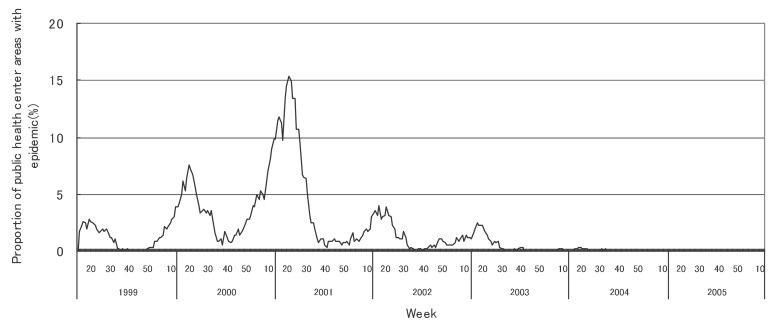
Proportion of public health center areas with epidemic of measles by week in fiscal 1999-2005.

**Figure 12.**
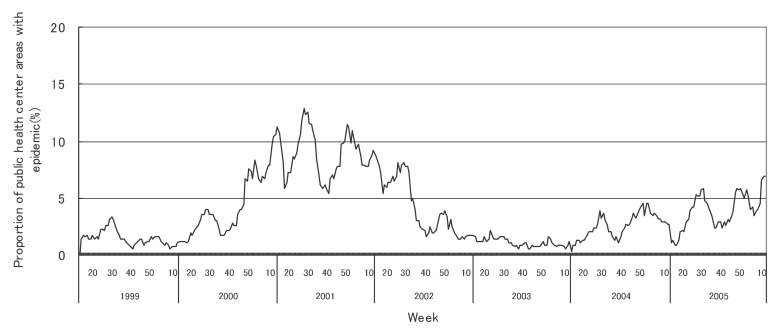
Proportion of public health center areas with epidemic of mumps by week in fiscal 1999-2005.

Table 4 shows the indices of temporal change of epidemic in influenza and 7 pediatric diseases excluding 4 diseases with low or no peak as observed in Figures 1 to 12. In influenza, the height of peak in the epidemic area proportions varied widely between 6 and 90% in the 7 years. The height of 90% means that the epidemic occurs at the week of the peak in 90% of the public health center areas across Japan. The week of the peak also varied widely. The durations of the elevation over the 5% level of the epidemic area proportion were 2 weeks in fiscal year of 2000, 14 weeks in 2002, and 7-9 weeks in other fiscal years. In pediatric diseases, the height of peak varied with the year, while the week of peak was relatively constant. In infectious gastroenteritis and herpangina, both the height and week of the peak were relatively constant. The durations of the elevation increased with the height of peak in most pediatric diseases, while low height of peak and relatively long duration were observed in the epidemic area proportion of hand-foot-mouth disease in 2004.

**Table 4.** Indices of temporal change of epidemic in fiscal 1999-2005.

Disease	Fiscal year						

	1999	2000	2001	2002	2003	2004	2005
Influenza							
Height of peak (%)^*^	59.5	5.5	25.7	66.7	59.5	90.1	55.4
Week of peak^*^	5th, 6th	11th, 12th	8th	5th	7th	10th	5th
First week^†, ‡^	3rd	11th	5th	52nd	4th	5th	2nd
Last week^†, ‡^	9th	12th	13th	13th	11th	13th	10th
Duration (in weeks) ^†^	7	2	9	14	8	9	9
Pharyngoconjunctival fever							
Height of peak (%)^*^	-	8.8	13.6	-	17.4	30.7	16.0
Week of peak^*^	-	34th	32nd	-	30th, 32nd	30th	33rd
First week^†, ‡^	-	28th	27th	-	22nd	17th	22nd
Last week^†, ‡^	-	38th	38th	-	41st	41st	43rd
Duration (in weeks)^†^	-	11	12	-	20	25	22
Infectious gastroenteritis							
Height of peak (%)^*^	39.8	34.5	33.6	23.2	36.8	34.7	38.5
Week of peak^*^	51st	51st	51st	49th	52nd	52nd	50th, 51st
First week^†, ‡^	47th	48th	47th	45th	47th	49th	47th
Last week^†, ‡^	2000.17th	13th	2002.14th	2003.14th	13th	10th	12th
Duration (in weeks)^†^	23	18	20	22	19	15	18
Chickenpox							
Height of peak (%)^*^	9.5	12.1	7.2	8.8	15.5	7.3	8.6
Week of peak^*^	1st	1st	2nd	2nd	2nd	1st	1st
First week^†, ‡^	50th	49th	51st	50th	48th	52nd	50th
Last week^†, ‡^	2nd	13th	2nd	2nd	5th	1st	2nd
Duration (in weeks)^†^	5	17	4	5	10	3	5
Hand-foot-mouth disease							
Height of peak (%)^*^	7.2	49.3	24.6	16.0	36.1	8.8	12.8
Week of peak^*^	29th, 30th	30th	29th	30th	31st	38th	28th
First week^†, ‡^	26th	21st	21st	25th	24th	28th	26th
Last week^†, ‡^	31st	48th	33rd	33rd	42nd	49th	33rd
Duration (in weeks)^†^	6	28	13	9	19	22	8
Erythema infectiosum							
Height of peak (%)^*^	-	6.5	21.5	13.4	-	12.4	8.8
Week of peak^*^	-	26th	26th	27th	-	22nd	25th
First week^†, ‡^	-	22nd	2001.3rd	2001.49th	-	16th	22nd
Last week^†, ‡^	-	28th	30th	30th	-	29th	29th
Duration (in weeks)^†^	-	7	28	34	-	14	8
Herpangina							
Height of peak (%)^*^	48.4	41.2	50.2	30.6	47.0	29.8	43.3
Week of peak^*^	30th	30th	30th	31st	31st	29th	28th, 29th
First week^†, ‡^	23rd	24th	25th	24th	25th	24th	24th
Last week^†, ‡^	38th	38th	36th	34th	39th	34th	36th
Duration (in weeks)^†^	16	15	12	11	15	11	13
Measles							
Height of peak (%)^*^	-	7.6	15.3	-	-	-	-
Week of peak^*^	-	20th	21st	-	-	-	-
First week^†, ‡^	-	17th	2001.6th	-	-	-	-
Last week^†, ‡^	-	23rd	31st	-	-	-	-
Duration (in weeks)^†^	-	7	26	-	-	-	-

* : The peak in proportions of public health center areas with epidemic in a given year.

† : First week, last week and duration over the 5% level of the proportion of public health center areas with epidemic.

‡ : Week preceding or following given fiscal year is mentioned if the elevation continued across the fiscal years.

## DISCUSSION

In influenza, the mean epidemic area proportion in the 7 years was 6.0%. It means that the epidemic occurred an average 3.1 weeks in a year in a public health center area. The seasonal pattern with one peak in a year was observed in the weekly epidemic area proportions, corresponding to the well-known characteristic of influenza.^16^ The height of peak in the weekly epidemic area proportions was 90% in a week of fiscal year of 2004. The epidemic in this year was regarded to be very large from the viewpoint of the number of cases reported. Our result suggested that the epidemic was widespread in Japan. The duration of the elevation over the 5% level of the epidemic area proportion was around 8 weeks in all but in 2000 with the small epidemic, against 14 weeks in fiscal year of 2002. The reason would be that a relatively large epidemic of type B virus followed after the epidemic of type A virus in the year.^17^

In pediatric diseases, the epidemic area proportion was low in rubella and pertussis, and decreased in measles. These results would be mainly associated with the vaccination program against these diseases in Japan. The vaccination coverage for rubella in children has been considered nearly complete as well as that for diphtheria pertussis tetanus vaccine.^18^ The coverage for measles recently increased with the well-developed vaccination program thanks to the amendment of the Immunization Law in Japan.^19^

The epidemic area proportion has increased in pharyngoconjunctival fever and group A streptococcal pharyngitis, possibly in connection with the introduction of rapid detection kits for their diagnosis. For example, although the criteria for reporting pharyngoconjunctival fever to the NESID in Japan include three principal symptoms (fever, pharyngeal rubor, and conjunctival hyperemia), kit detection of cases with upper respiratory tract inflammation without conjunctival hyperemia might result in incorrectly reported disease.

In many pediatric diseases, the seasonal pattern with one peak in a year was observed in the weekly epidemic area proportions as well as in influenza. This is consistent with the well-known findings.^16^ Two peaks in a year were observed in the weekly epidemic area proportions of infectious gastroenteritis. It could be explained by the two different agents: noroviruses for the former peak and rotaviruses for the latter.^20^

There are some limitations in the present study. One of the key issues is the determination of an epidemic, which can be done by several methods and is not yet standardized.^5^^,^^11^ The method used in the present study has been applied as an alert system for epidemics in the NESID in Japan since 1999. Certainly, determination of the frequency of epidemics depends heavily on the method employed, and is affected by the critical values in the method used. If the critical value for the onset of an epidemic was lowered, the frequency of epidemics and height of the epidemic peak increased, and the duration of the epidemic was lengthened. If the critical value for the end of the epidemic was lowered, the week of epidemic peak was delayed.^15^ However, the temporal change of epidemics might not change substantially according to some changes in the critical values. Other issues would be possible bias due to the use of data from reports to the NESID in Japan, such as accuracy of disease diagnosis, coverage of reporting and representativeness of sentinels for all medical institutions.

In conclusion, although there are some limitations, the present study is, to our knowledge, the first to report the frequency and temporal change in the epidemics of influenza and pediatric diseases in public health center areas from NESID data in Japan for the fiscal years of 1999-2005. Further analyses with mapping techniques are expected to provide additional information on the geographical and temporal spread of disease epidemics.
